# Notes and News

**Published:** 1897-05-01

**Authors:** 


### Notes and News.

(Collected and Communicated.)

The Blackwall Tunnel will be opened by the Prince and Princess of Wales on Saturday, May 22nd, not on May 12th as was mentioned last week.

The annual festival of the Royal Hospital for Incurables will be held at Merchant Taylors' Hall, Threadneedle Street, on Tuesday, June 1st.

New wards and a new operating room have been opened in the North Riding Infirmary at Middlesborough. A nurses' room, bath rooms, and lavatories have also been added.

The treasurer of Guy's Hospital has received £500 from Mr. Walter James, and a further donation of £500 from Mr. Thomas A. Mason in aid of the re-endowment fund.

Lord Rothschild has promised a donation of £200 towards the Jubilee celebration at Slough, through which town the Queen will pass on her return to Windsor, after June 21st. Very extensive decorations are being arranged all along the route.

The Huddersfield Infirmary has received the munificent gift of £8,000 from Mr. Hildred Carlile in commemoration of the Queen's long reign. This will enable the board of the infirmary to carry out all the additions and alterations necessary to render it complete and up-to-date.

After much discussion it has been resolved to appeal to the people of Glasgow for £100,000 to erect a New Royal Glasgow Infirmary on the same site as the old one. It is understood that the new building will be one storey higher than the present one, and will contain extra accommodation for about 120 patients, which will then render it possible to receive 720 inmates.

The Prince of Wales, whilst visiting Cannes, laid the foundation-stone of the new British Hospital there. The building will be connected with the old institution on the ground floor only. It will contain eight wards, a surgeon's room, large hall, and a dining-room for the staff. The lavatories have been carefully isolated. The heating will be by hot-water pipes. A separate building will contain four isolation wards with complete offices. Mr. E. Hewetson, an architect at Cannes, has designed the hospital, which is to cost 55,000 francs. The people of Cannes will, when the hospital is completed, be able to boast of an institution such as is possessed by no other Continental health resort.

Mr. Benjamin Cohen, M.P., will preside at the banquet to be given in aid of the Great Northern Central Hospital to commemorate the Queen's long reign, at the Hotel Cecil, on Tuesday, May 11th.

Dr. Felix Semon has resigned his post as physician to the throat department at St. George's Hospital. At the general meeting of the hospital Dr. Semon was warmly thanked for his valuable services to the institution.

Mr. Mackenzie, of Fawley Court, has promised a contribution of £1,000 to the British Orphan Asylum, provided other donors come forward to increase the reserve fund of the institution by a substantial sum amounting to about £20,000.

South of Poona the plague has nearly disappeared, whilst from Poona upwards of 213 deaths have occurred. In Bombay the number of cases and deaths shows a slight increase, as numbers of the population are flocking back again to their insanitary dwellings.

Four members of the Royal Commission on Tuberculosis, accompanied by Professor M'Fadyean, have gone to Brussels, Cologne, Leipzig, and Berlin to study the methods adopted in dealing with tuberculosis in meat.

We have received the prospectus of the Anti-Quackery League, a body to which we need hardly say we wish every success. The list of the committee is headed by the name of Sir Charles A. Cameron, a well-known sanitarian, and also contains those of Mr. Braxton Hicks, a well-known coroner, and of Dr. Gordon Stables, a very well-known writer, besides many others, so that it may be hoped that quackery will find itself assailed in many quarters.

The *Graphic* and *Daily Graphic* have issued a most patriotic notice, which runs as follows, in respect to a view of the Jubilee procession: To minimise the responsibility of deciding on the numerous claims of those interested in, and associated with, the *Graphic* and *Daily Graphic*, it has been decided as most appropriate and fitting to the occasion to give priority in allotting the seats to those most active in obtaining financial support to the Prince of Wales's Hospital Fund through the forms of the *Daily Graphic*. Illustrated collecting lists may be obtained from the secretary, and should be sent in filled up as soon as possible, but not later than the end of May.

We have frequently urged the advisability of some restrictive regulations in respect to the opening of new hospitals, private or otherwise, that patients and the public may be in some measure guarded from the dangers which attend inefficiently conducted and insanitary institutions. The Prussian Government realise the attendant dangers so fully that they have issued regulations containing conditions which render it almost impossible for private institutions not strictly conducted on hospital lines to exist at all. Court-yards, corridors, staircases, must conform to certain given measurements of height and width and conditions of ventilation, and north rooms are not permissible. In fact, such minute instructions are laid down that an agitation is on foot for the repeal of many of the restrictions when applied to those houses which admit patients for treatment or rest.

